# Nicotinamide riboside functions during development while beta-hydroxybutyrate functions during adulthood to extend *C. elegans* lifespan

**DOI:** 10.17912/micropub.biology.000841

**Published:** 2023-06-01

**Authors:** J. Dylan Peters, McKenzie P. Peters, Patrick C. Bradshaw

**Affiliations:** 1 James H. Quillen College of Medicine, East Tennessee State University, Johnson City, Tennessee, USA; 2 East Tennessee State University, Johnson City, Tennessee, USA; 3 Department of Biomedical Sciences, East Tennessee State University, Johnson City, Tennessee, USA

## Abstract

Nicotinamide riboside (NR), a form of vitamin B3 and a nicotinamide adenine dinucleotide (NAD ^+^ ) precursor, has been shown to activate the mitochondrial unfolded protein response (UPR ^mt^ ) and extend the lifespan when supplemented to *C. elegans.* The ketone body and histone deacetylase (HDAC) inhibitor beta-hydroxybutyrate (BHB) has also been shown to extend *C. elegans* lifespan. Experiments were performed that showed that NR extended lifespan by acting almost exclusively during larval development, while BHB extended lifespan by acting during adulthood, and the combination of NR during development and BHB during adulthood unexpectedly decreased lifespan. This suggests that hormesis is involved in the lifespan-altering effects of BHB and NR and that they are inducing parallel longevity pathways that converge on a common downstream target.

**Figure 1. The effects of NR, BHB, or both in combination on C. elegans lifespan when the compounds were present only during adulthood, during larval development and adulthood, or when NR was present only during larval development:**
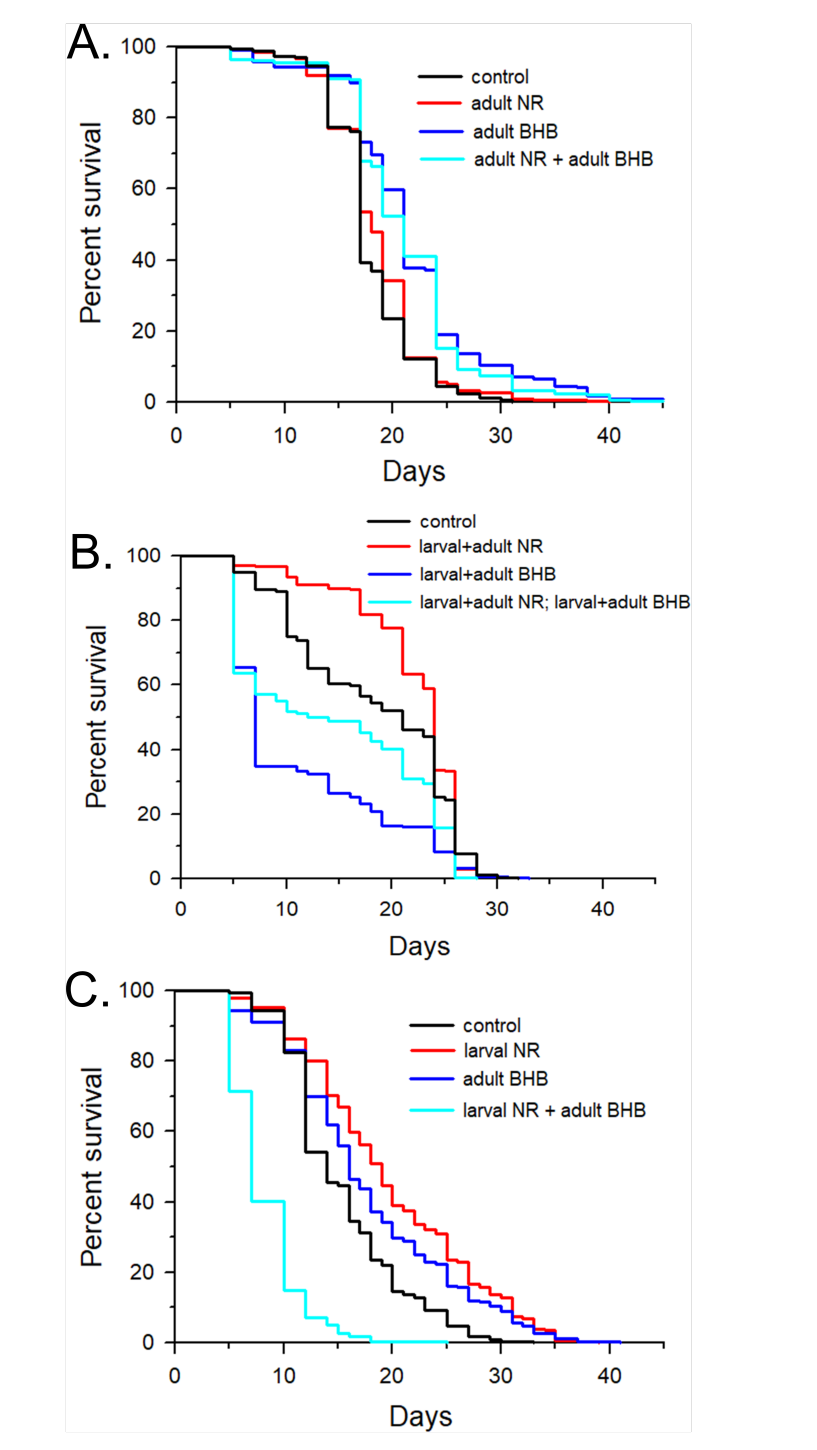
**A.** 0.5 mM NR, 20 mM BHB, both, or neither were added starting at the L4 larval stage. **B.** 0.5 mM NR, 20 mM BHB, both, or neither were added starting at the L1 larval stage. **C.** 0.5 mM NR was present only during the L1 to L4 larval stages, 20 mM BHB was present only from the L4 larval stage throughout adulthood, both treatments were combined, or no addition was made.

## Description

Nicotinamide adenine dinucleotide (NAD ^+^ ) is a coenzyme and electron acceptor that functions in roughly 300 human enzymatic reactions (Goodman et al., 2018) . Decreased levels of NAD ^+^ have been found in aged humans (Zhu et al., 2015) , mice (McReynolds et al., 2021) , fruit flies (Sohal et al., 1990) , and nematodes (Copes et al., 2015) potentially leading to decreased rates of metabolism and sirtuin deacetylase activity. Administering NAD ^+^ or one of its precursors nicotinamide riboside (NR) to nematodes extended their lifespan (Hashimoto et al., 2010; Mouchiroud et al., 2013) , while NR administration did not extend the lifespan of mice (Harrison et al., 2021) . However, administration of the more common NAD ^+^ precursor nicotinamide (NAM) was shown extend the health span of mice (Mitchell et al., 2018) and administration of NR or its phosphorylated derivative nicotinamide mononucleotide (NMN) can delay many aging-related pathologies (Gong et al., 2013) . Therapies that increase levels of the ketone body D-beta-hydroxybutyrate (D-BHB) have also been shown to increase the lifespan of mice (Newman et al., 2017; Roberts et al., 2017) , fruit flies (Fan et al., 2021) , and *C. elegans* (Edwards et al., 2014) . In mammals both endogenous hepatic ketogenesis or exogenous supplementation of BHB salts or ketone esters (Stubbs et al., 2017) can be utilized to induce nutritional ketosis (L. Wang et al., 2021). Increasing ketone body levels through consuming a ketogenic diet has been shown to increase the NAD ^+^ /NADH ratio in mouse brain hippocampus as a potential neuroprotective mechanism (Elamin et al., 2017; Elamin et al., 2020) . It has yet to be determined if NR or BHB are required during *C. elegans* larval development or adulthood for lifespan extension. Administration of NR to *C. elegans* leads to activation of the mitochondrial unfolded protein response (UPR ^mt^ ) (Mouchiroud et al., 2013) . When activated during larval development the UPR ^mt^ can extend lifespan (Dillin et al., 2002) , but activation of the UPR ^mt^ is not always associated with longevity (Bennett & Kaeberlein, 2014; Bennett et al., 2014) . When NR is administered during *C. elegans* adulthood, it induces a blunted version of the UPR ^mt^ associated with increased body wall muscle function and mitochondrial function and decreased muscle amyloidogenesis in older adulthood (Romani et al., 2021) .

The effects on *C. elegans* lifespan of treatment with 0.5 mM NR or 20 mM BHB or both together starting at the L4 larval stage of development and continuing throughout the lifespan is shown in **Figure 1A** . The results are summarized in Experiment 1 in **Table 1** . The concentrations of BHB and NR used were those shown to be most effective in previous lifespan studies (Edwards et al., 2014; Mouchiroud et al., 2013) . NR treatment only increased mean lifespan by 3%, while BHB increased mean lifespan by 21%. Combination treatment increased mean lifespan by 18% that was not significantly different than the addition of BHB alone.

Lifespan experiments were then performed using the same treatments, but they were first administered at the L1 larval stage ( **Figure 1B** ) instead of the L4 stage. The results are summarized in Experiment 2 in **Table 1.** NR increased mean lifespan by 20% consistent with previous results of NR-mediated longevity (Mouchiroud et al., 2013) and suggesting the requirement of the full non-blunted UPR ^mt^ activated during larval development for the anti-aging effects. Surprisingly, administration of 20 mM BHB decreased lifespan by 40% suggesting larval toxicity. When NR and BHB were administered together starting at the L1 stage, there was a 22% decrease in mean lifespan. Therefore, the presence of NR blunted the toxicity of BHB during larval development.

As shown in **Figure 1C** , lifespan experiments were then performed when administering *C. elegans* 0.5 mM NR only during the L1 to L4 larval stages, administering 20 mM BHB starting at the L4 stage and continuing throughout adulthood (as performed in Experiment 1 described above), or the combination of the previous two treatments. The results are described in Experiment 3 in **Table 1.** Treatment with NR only during larval development increased mean lifespan by 28%, while treatment with BHB only during adulthood increased mean lifespan by 16%, slightly less potently than the 21% observed under the identical conditions in Experiment 1. Lastly, the combined treatment of NR only present during the larval stages and BHB only present from the L4 stage onward surprisingly decreased mean lifespan by 46%, even though each individual treatment increased lifespan.

**Table:** 

**Treatment**	**Treatment duration**	**Mean survival (days)**	**Mean lifespan change**	**Log-rank** **p-value**	** # of replicates ( *n* ) **	**Worms counted**
**Experiment 1: Treatment during adulthood**						
Control		17.9		p < 0.001	3	529
NR	L4 onward	18.4	+3%	p = 0.008	3	428
BHB	L4 onward	21.6	+21%	p < 0.001	3	396
NR+BHB	L4 onward	21.0	+18%	p < 0.001	3	438
**Experiment 2: Treatment throughout development and adulthood**						
Control		18.4			3	318
NR	L1 onward	22.0	+20%	p = 0.001	3	251
BHB	L1 onward	11.0	-40%	p < 0.001	3	313
NR+BHB	L1 onward	14.4	-22%	p < 0.001	3	308
**Experiment 3: Combining the individual lifespan-extending treatments**						
Control		15.3			4	471
NR	L1 to L4	19.7	+28%	p < 0.001	4	531
BHB	L4 onward	17.7	+16%	p < 0.001	4	554
NR+BHB	NR L1-L4, BHB L4 onward	8.2	-46%	p < 0.001	4	495

**Table 1.** **The effects of NR, BHB, or both in combination on *C. elegans* lifespan.** The change in mean lifespan of *C. elegans* when 0.5 mM NR, 20 mM BHB, neither, or the combination of both treatments were present at different times throughout the lifespan as indicated.

In summary, the data show that NR functions during larval development to extend *C. elegans* lifespan, while BHB functions during adulthood to extend lifespan. The combination of the two compounds decreased lifespan suggesting that NR and BHB extend lifespan through parallel pathways that converge on common downstream targets. The common targets are likely the DAF-16 /FOXO and SKN-1 /Nrf2 longevity-inducing transcriptional regulators. NR (Mouchiroud et al., 2013) , NAD ^+^ (Hashimoto et al., 2010) , or BHB (Edwards et al., 2014) were all shown to require DAF-16 for lifespan extension. BHB was also shown to require SKN-1 for lifespan extension (Edwards et al., 2014) , while the UPR ^mt^ was shown to lead to SKN-1 activation as well (Soo et al., 2021) . However, complexly, NR and BHB administration had an additive effect on lifespan when both were present together during larval development, the time when BHB induced its toxic effect. This likely indicates that the toxic effect of BHB during development prevented the adulthood administration of BHB from activating the common longevity-promoting downstream target, which was therefore only activated by NR yielding the additive effect on lifespan. The different temporal requirements for NR and BHB-mediated lifespan extension suggest that BHB does not extend lifespan by increasing NAD ^+^ levels.

The effects of NR and BHB on longevity appear to be due to hormesis or mitohormesis (Cypser et al., 2006; Sun et al., 2020) . A lower concentration of 10 mM BHB, than the 20 mM dose used here, was shown to extend *C. elegans* lifespan when present throughout the lifespan starting from early larval development (Edwards et al., 2014) . This data together with the data shown here support a mechanism of hormesis. In the same report, it was also shown that BHB activated the SKN-1 transcriptional regulator, which is commonly activated by ROS suggesting a role for mitohormesis. Another common effector of mitohormesis is loss of the mitochondrial membrane potential during *C. elegans* larval development, which induces the translocation of the ATFS-1 transcriptional regulator from the mitochondria to the nucleus. This ATFS-1 nuclear translocation, as well as the nuclear translocation of the DVE-1 transcriptional regulator, induces the UPR ^mt^ that remodels chromatin in part by inducing the expression of histone H3K27me2/3 demethylases JMJD-1.2 and JMJD-3.1 (Merkwirth et al., 2016) to increase gene expression leading to increased mitochondrial protein import (Xin et al., 2022) and activation of stress response pathways that can extend lifespan (Soo et al., 2021) .

BHB has been shown to function as a weak class I histone deacetylase (HDAC) inhibitor to induce gene expression, including the expression of the DAF-16 homolog FOXO3A (Shimazu et al., 2013) . Low dose administration of class I HDAC inhibitors activate gene expression and extend lifespan in yeast, worms, and flies (McIntyre et al., 2019) , while higher doses have disparate effects, sometimes hyperactivating and sometimes repressing gene expression (Sanchez et al., 2018) , leading to toxicity and decreased lifespan (Edwards et al., 2014; Evason et al., 2008) . Consistent with this, knockdown of the expression of the *C. elegans* class I HDACs *hda-2* or *hda-3* extended lifespan, while complete deficiency of *hda-2* or *hda-3* decreased lifespan (Edwards et al., 2014) . Therefore, altered levels of histone acetylation likely explain why BHB administration during larval development, a time when gene expression needs to be tightly regulated, decreased lifespan.

There is competition on specific histone lysine residues, most notably on histone H3K27, between (tri)methylation that represses gene expression and acetylation that induces gene expression (Zhang et al., 2015) . Therefore, NR-induced activation of the UPR ^mt^ and induction of the expression and activity of histone H3K27me2/3 demethylases likely increases histone H3K27 acetylation. When combined with the further increased histone acetylation due to BHB-mediated class I HDAC inhibition, this likely leads to excessive histone H3K27 acetylation (Sanchez et al., 2018) and decreased lifespan, which mimics the decreased longevity of the *hda-2* and *hda-3* mutant strains (Edwards et al., 2014) .

The results of the experiments presented here suggest that NR does not likely extend lifespan by preventing the aging-related loss of NAD ^+^ , but instead by activating the UPR ^mt^ and inducing epigenetic changes during development. It remains to be determined if UPR ^mt^ activation prevents the loss of tissue NAD ^+^ levels with aging. The mechanism through which increased NAD ^+^ activates the UPR ^mt^ has also yet to be elucidated. However, a strong possibility is that it increases mitochondrial NAD ^+^ levels and possibly also cytoplasmic and mitochondrial NADP ^+^ levels to increase the activity of one or more of the three cellular NAD(P) ^+^ -dependent isocitrate dehydrogenase enzymes to increase the levels of alpha-ketoglutarate, an essential cofactor for several histone demethylases including JMJD-1.2 and JMJD-3.1 involved in UPR ^mt^ activation (Merkwirth et al., 2016) . The results from this report also suggest that it is of interest to determine if injecting NR into pregnant mice will lead to UPR ^mt^ activation in the progeny leading to their lifespan extension.

The experiments described here have several limitations, with the use of 5-fluorodeoxyuridine (FUdR) for nematode sterilization likely being the most important. Although FUdR has little effect on normal wild-type N2 lifespan (H. Wang et al., 2019), it has been shown to greatly increase the lifespan of some mutant strains (Aitlhadj & Stürzenbaum, 2010; Van Raamsdonk & Hekimi, 2011). Another limitation is the use of DL-BHB when D-BHB is the lifespan extending isomer (Edwards et al., 2014) . It was not determined if D-BHB or L-BHB was the toxic agent leading to the decreased lifespan.

In conclusion, NR robustly extended lifespan when it was administered only during *C. elegans* larval development, and BHB robustly extended lifespan when it was administered only during adulthood. The combination greatly decreased lifespan suggesting that hormesis plays a role in the lifespan extension and that the two compounds activate parallel longevity pathways *.*

## Methods

***C. elegans* culture.**

A mixed age population of wild-type N2 *C. elegans* was cultured on nematode growth media (NGM) agar plates according to standard practice (Stiernagle, 2006) . Once a sufficient amount of *C. elegans* was obtained, an age synchronization protocol was performed as described in (Stiernagle, 2006) . Briefly, this entailed washing the NGM agar plates containing *C. elegans* with a chilled 0.1 M NaCl solution. The suspension of *C. elegans* nematodes in 0.1 M NaCl was then transferred to 50 mL conical tubes. Following this step, the worms were suspended in a 1% bleach and 0.5 M NaOH solution. This solution killed any larval or adult *C. elegans* contained in the suspension, but not the eggs, which are protected by their eggshell (Stiernagle, 2006) . This procedure ensured that the eggs collected and utilized in the lifespan assay were age-synchronized within 9 hours.

**Lifespan assay protocol.**

Following this procedure, the eggs were placed in M9 minimal growth media overnight. In this solution, the nematodes were able to hatch into the L1 larval stage, but they could not develop further until they were fed. The next day the nematodes were spun down and resuspended in liquid S-medium and 25-60 nematodes in a volume of 1.35 mL were transferred into an 8-micron cell culture insert, which was placed into a well of a 12-well microplate containing 0.15 mL of 9 x 10 ^9^ (10 mg protein/mL) HT115 (DE3) *E. coli* cells/mL. The *E. coli* were grown in LB media, spun down, and resuspended. To provide complete nutrition during the larval stages, the worms were fed live *E. coli* through the first two days of the experiment. The microplates, with a total volume of 1.5 mL each, were incubated at 20°C and shaken at 60 rpm to allow aeration. On the third day, and every Monday, Wednesday, and Friday following, the cell culture media was replaced with media containing heat-killed *E. coli* of the same concentration and strain. The heat killing prevents the bacteria from metabolizing the treatment compounds. The *E. coli* were killed by incubating them at 80 °C for two hours. To change out the culture media the inserts were first raised out of the wells. The media in the wells was then aspirated off and the media remaining in the cell culture inserts was removed by capillary action by touching a chemical wipe tissue to the bottom side of the insert. On the third day when the nematodes reached the L4 stage, 0.4 mM FUdR was added to each well. FUdR is a DNA synthesis inhibitor, which kills the actively dividing germ cells sterilizing the nematodes, allowing for a synchronous nematode population throughout the duration of the experiment. Every Monday, Wednesday, and Friday when the *C. elegans* were adults, the number of live worms in each insert was counted under a stereomicroscope, and the culture medium containing bacteria, FUdR and treatment compound was replaced. Upon the death of all *C. elegans* in each set of experiments, the mean lifespans and percent change in mean survival compared to the untreated controls were calculated using Log-rank statistical analysis and graphed using Kaplan-Meier survival curves. Significant changes in lifespan were defined as Log-rank p < 0.05.

**Lifespan assay treatments.**

For each of the three sets of lifespan experiments one cohort of worms was treated with 0.5 mM nicotinamide riboside chloride (NR), while the second cohort was treated with 20 mM sodium DL-BHB (BHB), while the third cohort was treated with both 0.5 mM NR and 20 mM BHB, and the fourth cohort was left untreated. The timing of compound administration differed among the three different sets of lifespan experiments. For the first set of experiments on day one of the experiment corresponding to the L1 larval phase, nematodes were administered NR, BHB, both compounds together, or were left untreated. The culture medium including treatment compounds were replaced every Monday, Wednesday, and Friday starting from the L4 stage when FUdR was introduced and continuing throughout the entirety of the lifespan. For the second set of lifespan experiments on the third day of the experiment when the nematodes were at the L4 larval stage, they were administered NR, BHB, both compounds together, or were left untreated. Culture media including the treatment compounds and FUdR were replaced each Monday, Wednesday and Friday continuing throughout the entirety of adulthood. In the last set of lifespan experiments one cohort of nematodes was administered 0.5 mM nicotinamide riboside only on day one of the experiment, so it was present only during the L1 to L4 larval stages, one cohort was treated with 20 mM BHB beginning from the L4 larval stage on every Monday, Wednesday, and Friday for the entirety of adulthood when the culture media was changed out. The third cohort of nematodes was treated with both 0.5 mM NR from the L1 to L4 larval stages and 20 mM BHB from the L4 larval stage throughout adulthood. The fourth cohort of nematodes was left untreated.

## Reagents

The *C. elegans* N2 strain and *E. coli* strain HT115 (DE3) were obtained from the Caenorhabditis Genetics Center (CGC) at the University of Minnesota, MN USA.

Nicotinamide riboside chloride was purchased from Medkoo Biosciences, Cary, NC USA.

Sodium DL-BHB (Prod. # 54965) was purchased Sigma-Aldrich, St. Louis, MO USA

12-well plate 8-micron cell culture inserts were purchased from VWR (product # 10769-218).

All solutions including NGM agar and liquid S-medium were prepared using analytical grade reagents.
